# Complementary and alternative medicine: attitudes, knowledge and use among surgeons and anaesthesiologists in Hungary

**DOI:** 10.1186/s12906-016-1426-0

**Published:** 2016-11-08

**Authors:** Sándor Árpád Soós, Norbert Jeszenői, Katalin Darvas, László Harsányi

**Affiliations:** 1 1st Department of Surgery, Semmelweis University, Budapest, Hungary; 2 MTA NAP-B Molecular Neuroendocrinology Group, Institute of Physiology, Szentágothai Research Centre, Centre for Neuroscience, University of Pécs, Pécs, Hungary; 3 Department of Genetics, Eötvös Loránd University, Budapest, Hungary; 4 Department of Anaesthesiology and Intensive Therapy, Semmelweis University, Budapest, Hungary

**Keywords:** Anaesthesiologist, Surgeon, CAM, Perioperative care, Integrative medicine

## Abstract

**Background:**

Despite their worldwide popularity the question of using non-conventional treatments is a source of controversy among medical professionals. Although these methods may have potential benefits it presents a problem when patients use non-conventional treatments in the perioperative period without informing their attending physician about it and this may cause adverse events and complications. To prevent this, physicians need to have a profound knowledge about non-conventional treatments.

**Methods:**

An anonymous questionnaire was distributed among surgeons and anaesthesiologists working in Hungarian university clinics and in selected city or county hospitals. Questionnaires were distributed by post, online or in person. Altogether 258 questionnaires were received from 22 clinical and hospital departments.

**Results:**

Anaesthesiologists and surgeons use reflexology, Traditional Chinese Medicine, herbal medicine and manual therapy most frequently in their clinical practice. Traditional Chinese Medicine was considered to be the most scientifically sound method, while homeopathy was perceived as the least well-grounded method. Neural therapy was the least well-known method among our subjects.

Among the subjects of our survey only 3.1 % of perioperative care physicians had some qualifications in non-conventional medicine, 12.4 % considered themselves to be well-informed in this topic and 48.4 % would like to study some complementary method. Women were significantly more interested in alternative treatments than men, *p* = 0.001427; OR: 2.2765. Anaesthesiologists would be significantly more willing to learn non-conventional methods than surgeons. 86.4 % of the participants thought that non-conventional treatments should be evaluated from the point of view of evidence. Both surgeons and anaesthesiologists accept the application of integrative medicine and they also approve of the idea of teaching these methods at universities.

**Conclusions:**

According to perioperative care physicians, non-conventional methods should be evaluated based on evidence. They also expressed a willingness to learn about those treatments that meet the criteria of evidence and apply these in their clinical practice.

**Electronic supplementary material:**

The online version of this article (doi:10.1186/s12906-016-1426-0) contains supplementary material, which is available to authorized users.

## Background

In recent decades, complementary and alternative medicine (CAM) has become increasingly popular. In the USA, the use of CAM rose from 33.8 to 42.1 % from 1990 to 1997 among the total population [1], while in the 2000s this ratio was 34–37 % [2, 3]. A recent systematic review of surveys in the UK indicated, 51.8 % of the population applied alternative remedies at least once during their lifetime [4]. Among cancer patients the use of CAM grew from 25 % in the 1970s to 49 % in the 2000s [5], and this rate can reach 61 % among orthopaedic cancer patients [6]. In Canada, 27 % of patients waiting for general surgery applied CAM [7], while in the USA the application ratio was 40–67 % [8, 9].

In Norway some forms of CAM treatment were available for patients in 50.5 % of hospitals in 2008 and in 64.4 % in 2013 [10]. Integrative medicine, which uses a holistic approach and CAM has grown popular not only among patients but also among medical professionals as it has gained more developed research basis [11].

The large-scale application of non-conventional treatments affect emergency care and the perioperative period [12]. Because of the risks of non-conventional treatments especially the potentially serious side-effects and drug interactions caused by herbal drugs, physicians working in perioperative care must be aware whether their patients apply CAM [13–15]. The situation regarding CAM methods is further complicated by the fact that if patients use them, they rarely inform their attending physician about it [16, 17]. The main reasons for this are past negative experiences of patients or their fear of doctor preconceptions about CAM [18].

In view of the above, we conducted a survey of the attitudes and knowledge of Hungarian physicians, surgeons and anaesthesiologist working in perioperative care.

## Methods

Our goal was to assess attitude of anaesthesiologists and surgeons towards CAM, their knowledge of particular treatments and to establish the extent to which they accept integrative medicine.

### Study design and data collection

The survey was conducted via a questionnaire distributed online, by mail or in person among anaesthesiologists and surgeons working in Hungary. Our survey is a part of a complex study focusing on the relationship between perioperative care and CAM.

Previous publications were focused on the herb consumption of patients waiting for elective surgery [19] and the attitude of surgery patients towards CAM [20]. In the near future, we are going to survey the attitude towards CAM of healthcare professionals working in perioperative care as well.

In the survey 11 clinics from 4 Hungarian universities and other 11 surgery wards and intensive care departments participated. The questionnaire was not validated.

### Questionnaire

The questionnaire consisted of four parts. The first part included socio-demographic data (age, gender, specialization, qualifications). In the second part, we enlisted those CAM methods that can be studied in Hungary and that were included in the 2010 position statement of the Hungarian Academy of Sciences. We used a table format to organize our data and we also examined how these methods were perceived by physicians [21]. In the third part, we explored those factors that influence subjects’ attitudes towards CAM. In the fourth part, yes/no questions were used to address some issues regarding integrative medicine, such as its application, and training and teaching opportunities in this field. The English translation of the questionnaire is available in Additional file 1.

### Statistical analysis

IBM SPSS 20.0 software *(SPSS Inc., Chicago IL)* was used to analyse data. We examined socio-demographic data using descriptive statistics. Independent-Samples T Test and Chi-squared test were applied to compare the opinion of anaesthesiologists and surgeons. Significant result was accepted as *p* < 0.05.

## Results

We processed 258 questionnaires: out of the 341 questionnaires we got 217 back in person or by mail (response rate 64 %) and 41 questionnaires were sent back to us online. Response ratio was not calculated for questionnaires received by mail as the number of physicians reached online was unknown (Additional file 2).

### Socio-demographic data

Our subjects included 151 males and 107 females, with an average age of 39.9 years (24–71 years; SD 12.078). Table 1 shows the distribution of physicians based on their qualifications and specialization. 49 subjects (19 %) had at least another qualifying exam. Eight subjects (3.1 %) had qualifications in naturopathy: 1 surgeon and 7 anaesthesiologists.

**Table 1:** Qualifications, gender distribution and specialisation of the physicians

		Surgeon (*n* = 83)	Anaesthesiologist (*n* = 175)	Total (*n* and %)
Qualifications	Post-secondary degree	21	5	26 (10.0)
	Specialist	41	102	143 (55.5)
	Resident	21	68	89 (34.5)
Gender	Male	71	80	151 (58.5)
	Female	12	95	107 (41.5)
Has other specialist qualifications?	Yes	23	26	49 (19.0)
	No	60	149	209 (81.0)

Almost half of the responses were from the capital city, Budapest (49.2 %) (Table 2).

**Table 2:** Geographic distribution of respondents

County	Surgeon (*n* = 83)	Anaesthesiologist (*n* = 175)	Total (*n* and %)
Budapest (capital)	61	66	127 (49.2)
Bács-Kiskun	7	12	19 (7.4)
Baranya	0	1	1 (0.4)
Borsod-Abaúj-Zemplén	0	20	20 (7.8)
Csongrád	0	4	4 (1.6)
Győr-Moson-Sopron	4	1	5 (2.0)
Hajdú-Bihar	0	17	17 (6.5)
Heves	1	0	1 (0.4)
Komárom-Esztergom	9	5	14 (5.4)
Pest	1	11	12 (4.6)
Szabolcs-Szatmár	0	22	22 (8.5)
Veszprém	0	1	1 (0.4)
Zala	0	15	15 (5.8)

### Evaluation of the treatments

The evaluation of individual treatments is presented in Table 3. The least known method was neural therapy (213 subjects; 82.6 %). The method considered to be the least scientifically well-grounded was homeopathy (161 subjects; 62.4 %), while Traditional Chinese Medicine (TCM, 149 subjects; 57.8 %) and manual therapy (84 subjects, 32.6 %) were perceived as the most scientifically acceptable treatments. The most frequently recommended methods were herbal medicine/phytotherapy (26 subjects, 10.1 %), TCM and manual therapy (22–22 subjects; 8.5–8.5 %), while reflexology resulted to be the most frequently applied method (6 subjects; 2.3 %). Anaesthesiologists recommended or applied TCM significantly more often than surgeons, *p* = 0.017555; OR: 4.0351 (95 % CI 1.1756–13.8496).

**Table 3:** Attitudes towards surveyed CAM methods

	Specialization	Does not know it (*n* and %)	Does not consider it to be scientifically well-grounded (*n* and %)	Considers it to be scientifically well-grounded (*n* and %)	Recommends it to patients (*n* and %)	Applies it (*n* and %)
TCM	Surgeon	16 (19.)	14 (16.9)	50 (60.2)	3 (3.6)	0 (0.0)
	Anaesthesiologist	33 (18.9)	20 (11.4)	99 (56.6)	19 (10.8)	4 (2.3)
	Total	49 (19.0)	34 (13.2)	149 (57.8)	22 (8.5)	4 (1.5)
Homeopathy	Surgeon	11 (13.2)	57 (68.7)	15 (18.1)	0 (0.0)	0 (0.0)
	Anaesthesiologist	27 (15.4)	104 (59.4)	31 (17.7)	12 (6.9)	1 (0.6)
	Total	38 (14.7)	161 (62.4)	46 (17.8)	12 (4.7)	1 (0.4)
Herbal medicine	Surgeon	34 (41.0)	17 (20.5)	21 (25.3)	9 (10.8)	2 (2.4)
	Anaesthesiologist	84 (48.0)	20 (11.4)	54 (30.9)	17 (9.7)	0 (0.0)
	Total	118 (45.7)	37 (14.3)	75 (29.1)	26 (10.1)	2 (0.2)
Manual therapy	Surgeon	33 (39.8)	18 (21.7)	27 (32.5)	5 (6.0)	0 (0.0)
	Anaesthesiologist	86 (49.1)	14 (8.0)	57 (32.6)	17 (9.7)	1 (0.6)
	Total	119 (46.1)	32 (12.4)	84 (32.6)	22 (8.5)	1 (0.4)
Neural therapy	Surgeon	69 (83.2)	8 (9.6)	4 (4.8)	2 (2.4)	0 (0.0)
	Anaesthesiologist	144 (82.3)	13 (7.4)	14 (8.0)	3 (1.7)	1 (0.6)
	Total	213 (82.6)	21 (8.1)	18 (7.0)	5 (1.9)	1 (0.4)
Reflexology	Surgeon	38 (45.8)	22 (26.5)	17 (20.5)	5 (6.0)	1 (1.2)
	Anaesthesiologist	82 (46.8)	34 (19.4)	46 (26.3)	8 (4.6)	5 (2.9)
	Total	120 (46.5)	56 (21.7)	63 (24.4)	13 (5.1)	6 (2.3)
Bioenergetic medicine	Surgeon	40 (48.2)	39 (47)	4 (4.8)	0 (0.0)	0 (0.0)
	Anaesthesiologist	94 (53.7)	62 (35.4)	15 (8.6)	4 (2.3)	0 (0.0)
	Total	134 (51.9)	101 (39.2)	19 (7.4)	4 (1.5)	0 (0.0)
Kinesiology	Surgeon	37 (44.6)	30 (36.1)	10 (12.1)	6 (7.2)	0 (0.0)
	Anaesthesiologist	93 (53.1)	35 (20.0)	36 (20.6)	11 (6.3)	0 (0.0)
	Total	130 (50.4)	65 (25.2)	46 (17.8)	17 (6.6)	0 (0.0)

### Attitudes towards CAM methods

Table 4 shows the extent to which subjects’ opinion about CAM was influenced by different factors. The most important factor was evidence, while the least important factor was the media. There was no significant difference between surgeons and anaesthesiologists in the evaluation of the factors listed.

**Table 4:** Factors influencing attitudes towards CAM

	Specialization	Significant influence (*n* and %)	Moderate influence (*n* and %)	No influence (*n* and %)
Evidence	Surgeon	75 (90.4)	6 (7.2)	2 (2.4)
	Anaesthesiologist	148 (84.6)	21 (12.0)	6 (3.4)
	Total	223 (86.4)	27 (10.5)	8 (3.1)
Colleagues’ opinion	Surgeon	25 (30.1)	44 (53.0)	14 (16.9)
	Anaesthesiologist	58 (33.1)	85 (48.6)	32 (18.3)
	Total	83 (32.2)	129 (50)	46 17.8)
Personal experience (own or that of a family member)	Surgeon	39 (47.0)	27 (32.5)	17 (20.5)
	Anaesthesiologist	91 (52.0)	59 (33.7)	25 (14.3)
	Total	130 (50.4)	86 (33.3)	42 (16.3)
Media	Surgeon	1 (1.2)	17 (20.5)	65 (78.3)
	Anaesthesiologist	4 (2.3)	36 (20.6)	135 (77.1)
	Total	5 (1.9)	53 (20.6)	200 (77.5)
More training opportunities	Surgeon	20 (24.1)	40 (48.2)	23 (27.7)
	Anaesthesiologist	54 (30.9)	77 (44.0)	44 (25.1)
	Total	74 (28.7)	117 (45.3)	67 (26.0)
Eligibility for reimbursement	Surgeon	6 (7.2)	34 (41.0)	43 (51.8)
	Anaesthesiologist	24 (13.7)	66 (37.7)	85 (48.6)
	Total	30 (11.6)	100 (38.8)	128 (49.6)

Table 5 shows attitudes towards CAM. No significant difference was found between the surgeons and anaesthesiologists, using the Independent-Samples T Test, 0.247; results were similar both in terms of interest (*p* = 0.462433), and in rejection (*p* = 0.177313). As for gender differences, the interest in CAM was significantly higher among women, *p* = 0.001427; OR: 2.2765 (95 % CI: 1.3674–3.7901), while the number of those who have a negative attitude towards these methods was significantly higher among men, *p* = 0.001015; OR: 4.6439 (95 % CI: 1.7305–12.4619).

**Table 5:** Evaluation of attitude towards CAM with respect to specialization and gender

	Interested (*n* and %)	Indifferent (*n* and %)	Negative (*n* and %)
Surgeon (*n* = 83)	41 (49.4)	28 (33.7)	14 (16.9)
Anaesthesiologist (*n* = 175)	95 (54.3)	61 (34.8)	19 (10.9)
Male (*n* = 151)	67 (44.4)	56 (37.1)	28 (18.5)
Female (*n* = 107)	69 (64.5)	33 (30.8)	5 (4.7)

**Fig. 1:**
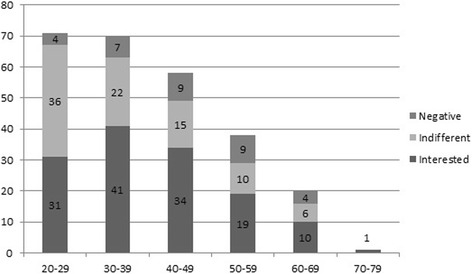
Attitudes towards CAM with respect to age

Perioperative physicians attitudes towards CAM with respect to age groups are shown in Fig. 1. In terms of interest no significant differences were found among the groups.

### Training and teaching possibilities in CAM and the application of integrative medicine

Further pragmatic aspects of the study can be seen in Table 6 (use of CAM, knowledge of this topic, education, acquisition, application, integrative treatment, reimbursement). 36 % (93 subjects) claimed to have used CAM in the case of their own illness or that of a family member. However, only 12.4 % (32 subjects) claimed to have enough knowledge about these methods. Almost half of the subjects would like to learn some CAM method, with anaesthesiologists being significantly more interested in this than surgeons, *p* = 0.000425; OR: 2.6397 (95 % CI 1.5265–4.5648). 6 surgeons and 12 anaesthesiologists, altogether 18 physicians (7 %) applies CAM in clinical practice.

**Table 6:** The use of CAM, evaluation of knowledge, integration into education and reimbursment

	Specialization	Yes (*n* and %)	No (*n* and %)	Independent-Samples T Test (t)	Pearson (*p*)
Uses CAM for their own or family member’s illnes	Surgeon	25 (30.1)	58 (69.9)	1.393	0.165
	Anaesthesiologist	68 (38.9)	107 (61.1)		
	Total	93 (36.0)	165 (64.0)		
Has enough knowledge about CAM	Surgeon	13 (15.7)	70 (84.3)	−1.032	0.304
	Anaesthesiologist	19 (10.9)	156 (89.1)		
	Total	32 (12.4)	226 (87.6)		
Would like to learn about CAM	Surgeon	27 (32.5)	56 (67.5)	3.669	0.000425
	Anaesthesiologist	98 (56.0)	77 (44.0)		
	Total	125 (48.4)	133 (51.6)		
CAM should be taught at universities	Surgeon	59 (71.1)	24 (28.9)	0.641	0.522
	Anaesthesiologist	131 (74.9)	44 (25.1)		
	Total	190 (73.6)	68 (26.4)		
Applies CAM in clinical practice	Surgeon	6 (7.2)	77 (92.8)	−0.109	0.913
	Anaesthesiologist	12 (6.9)	163 (93.1)		
	Total	18 (7.0)	240 (93.0)		
Integrative treatment	Surgeon	51 (61.4)	32 (38.6)	1.200	0.232
	Anaesthesiologist	121 (69.1)	54 (30.9)		
	Total	172 (66.7)	86 (33.3)		
Integrated reimbursement	Surgeon	28 (33.7)	55 (66.3)	2.743	0.007708
	Anaesthesiologist	90 (51.4)	85 (48.6)		
	Total	118 (45.7)	140 (54.3)		

Two-thirds of participant supported the concept of integrative medicine and in this aspect, no significant differences was found, *p* = 0.232.

A significantly higher number of anaesthesiologists than surgeons supported the idea that CAM should be an integral part of modern medicine in terms of reimbursement, accessible via the public healthcare system in Hungary, *p* = 0.007708; OR: 2.0798 (95 % CI 1.2083–3.58). 73.6 % (190 subjects) think that training in CAM should be completed within the frameworks of education at medical universities. This was considered to be important both by surgeons and anaesthesiologists, there were no significant differences between the two groups in this aspect, *p* = 0.522.

## Discussion

### Use of CAM in clinical practice

Our primary aim was to measure how frequently CAM is used by perioperative physicians in Hungary. Surveys concerning the attitude towards CAM among surgeons and anaesthesiologists were not found. Conducted studies emphasize on family physicians or physicians working on other fields. In England, 32 % of hospital physicians have already used CAM in their practice [22], this ratio is 24 % in the USA and 58 % in India [23, 24]. In Germany 31 % of orthopaedic surgeons, 23 % of family physicians and 6 % of internists apply CAM [25]. In our survey, 18 surgeons and anaesthesiologists (7 %) claimed to use CAM: this number is much lower than the average rate of CAM-usage among hospital physicians, as can be found in the corresponding literature.

### Attitude and predictors

Our survey examined the acceptance of CAM and its predictors. CAM is applied by a well-defined group: among the patients significantly more females, with high income and education use CAM, and application increases with serious clinical conditions [26, 27]. However, predictors of physicians applying or recommending CAM were seldom analysed. According to a recently published Polish survey, eldery physicians were more positive towards CAM than their younger colleagues. [28]. According to a German survey, 51 % of physicians were in favor of CAM use in clinical practice: it is not influenced by the age or the gender of the physicians. However, compared to other physicians, family physicians are more likely to apply CAM [29]. In our survey, more than half of the participants (52.7 %) expressed interest. Two main factors influencing their opinion were identified. Though only 32 physicians (12 %) have enough knowledge about CAM, scientific evidence was cited by 86 % of the participants as significantly influential. Personal experience was an important factor too as 93 physicians (36 %) have used CAM for their own or family member’s illness. Women showed significantly more interest than men, similarly to patients. Attitudes towards CAM were not influenced by age, and no significant differences were found between surgeons and anaesthesiologists.

### The evaluation of individual methods

The acceptance of CAM methods was also examined in the survey. In an Italian study, 8 % of those surveyed physicians use CAM and the most widely accepted methods are homeopathy, TCM, phytotherapy and manual therapy. These physicians are specialized in musculoskeletal apparatus or anaesthesiology [30]. A survey in Torino found that 84.2 % of primary care physicians consider TCM to be a scientifically well-based method and 6 % apply it in practice [31]. According to German, English and Swiss surveys carried out amongst family physicians, the most widely accepted methods are TCM, manual therapy, homeopathy, phytotherapy and neural therapy [32–34]. In our study we found that the most popular methods include TCM, herbal medicine/phytotherapy, manual therapy and reflexology, while homeopathy is the most controversial and neural therapy the least known method.

### Willingness to learn

Surveying the willingness to learn CAM methods is crucial as it represents openness and positivity towards CAM. As far as training opportunities in CAM methods are concerned, 55.8 % of healthcare professionals would like to learn about CAM according to a Swedish survey [35], while in England this number is 56 % [36]. In Hungary 56.7 % of family physicians would like to study CAM [37]. Our own findings reveal that 48.4 % of those surveyed would like to learn CAM: 32.5 % of surgeons and 56.0 % of anaesthesiologists, which is a significant difference. Thus similar trends observed in other countries can be observed in Hungary as well, anaesthesiologists have shown more openness towards CAM than surgeons. 73.6 % of our subjects considers it to be important that these methods should be taught in university settings.

### Integrative medicine

Integrative medicine - the combined use of modern and alternative treatments - is becoming more widespread within primary care [38], gynaecology [39], palliative medicine [40], oncology [41]. In an Italian survey, 58 % of primary care physicians advised their patients to use CAM [42], while in a Russian survey, 100 % of physicians may recommend these treatments [43]. Some suggest that it would be useful if physicians could acquire this new approach as part of their training [44, 45]. In our survey, two-thirds of surgeons and anaesthesiologists supported the introduction of integrative medicine. The issue of financial support was considered to be significantly more important by anaesthesiologists.

## Conclusions

Our survey was aimed at the assessment of surgeons’ and anaesthesiologists’ attitude towards CAM methods and their application, instruction and integration. Even though, compared with other specializations, the rate of CAM qualifications is low in the examined group of physicians, our results reveal that perioperative care physicians find the application of evidence-based CAM methods acceptable both in their private life and in their clinical practice.

## Additional files

Additional file 1: The English translation of the questionnaire. (DOCX 18 kb)
Additional file 2: List of hospitals participated in the survey. (DOCX 19 kb)

## Abbreviations

CAM: Complementary and Alternative Medicine
CI: Confidence interval
OR: Odds ratio
TCM: Traditional Chinese Medicine
