# Supplementary material for: Complementary and alternative medicine: attitudes, knowledge and use among surgeons and anaesthesiologists in Hungary
Source: BMC Complement Altern Med. 2016 Nov 8;16:443. doi: 10.1186/s12906-016-1426-0 (PMC5100213; doi:10.1186/s12906-016-1426-0)
Supplement: Additional file 1: — The English translation of the questionnaire. (DOCX 18 kb) [file 12906_2016_1426_MOESM1_ESM.docx]

Dear Colleague,

please, help our research, conducted in the Doctoral School of Clinical Medicine of the Semmelweis University, by completing the following questionnaire.

The questions of this questionnaire concern non-conventional therapies. We would like to assess your knowledge and opinion about the subject whether do you think these methods have role in health care.

The completing of the questionnaire is voluntary and anonymous. If you decide to complete the questionnaire, please be aware, that your positive or negative opinions are both very important for us. There are no right or wrong answers. Please do not miss a single issue.

If you complete the questionnaire, please answer all the questions!

We are grateful for your contribution!

Yours faithfully

Sándor Soós MD

Semmelweis University

1st Department of Surgery

**Questionnaire concerning the knowledge and usage of non-conventional therapies, and their possible applications in perioperative care**

**Age**………

**Sex**: male ⁯ female ⁯

**Workplace:**

Budapest ⁯ County:……………….

Surgeon ⁯ Anaesthesiologist ⁯

Post-secondary degree ⁯ Specialist ⁯ Resident ⁯

In the following tables CAM methods available in Hungary are specified. Please mark the answer with an X, you feel most suitable. There are no right or wrong answers. Please do not miss a single issue.

**What is your opinion regarding the following non-conventional methods?**

|  | **Does not know it** | **Does not consider it to be scientifically well-grounded** | **Considers it to be scientifically well-grounded** | **Recommends it to patients** | **Applies it** |
| --- | --- | --- | --- | --- | --- |
| Traditional Chinese Medicine (TCM) |  |  |  |  |  |
| Homeopathy |  |  |  |  |  |
| Herbal medicine |  |  |  |  |  |
| Manual therapy |  |  |  |  |  |
| Neural therapy |  |  |  |  |  |
| Reflexology |  |  |  |  |  |
| Bioenergetic medicine |  |  |  |  |  |
| Kinesiology |  |  |  |  |  |

**Which factors influence your opinion about naturopathy?**

|  | **Significant influence** | **Moderate influence** | **No influence** |
| --- | --- | --- | --- |
| Evidence |  |  |  |
| Colleagues’ opinion |  |  |  |
| Personal experience (own or that of a family member) |  |  |  |
| Media |  |  |  |
| More training opportunities |  |  |  |
| Eligibility for reimbursement |  |  |  |

**Do you have other specialist qualifications?**

Yes ⁯ No ⁯

**Do you have qualifications in CAM?**

Yes ⁯ No ⁯

**Do you apply CAM in clinical practice?**

Yes ⁯ No ⁯

**Are you interested in learning CAM?**

Yes ⁯ No ⁯

**Do you use CAM in for your own or family member's illness?**

Yes ⁯ No ⁯

**How do you evaluate your attitude towards CAM?**

Interested ⁯

Indifferent ⁯

Negative ⁯

**Do you have enough knowledge about CAM?**

Yes ⁯ No ⁯

**Do you think CAM should be integrated in medical care...**

**...as integrative treatment?**

Yes ⁯ No ⁯

**...as integrative reimbursement?**

Yes ⁯ No ⁯

**Do you think that CAM should be taught in the universities??**

Yes ⁯ No ⁯
