# Supplementary material for: Complementary and alternative medicine: attitudes, knowledge and use among surgeons and anaesthesiologists in Hungary
Source: BMC Complement Altern Med. 2016 Nov 8;16:443. doi: 10.1186/s12906-016-1426-0 (PMC5100213; doi:10.1186/s12906-016-1426-0)
Supplement: Additional file 2: — List of hospitals participated in the survey. (DOCX 19 kb) [file 12906_2016_1426_MOESM2_ESM.docx]

**By mail or by person:**

| **Hospitals** | **Sent**  **(n=299+42)** | **Received (n=217)** |
| --- | --- | --- |
| Bács-Kiskun Megyei Kórház, ICU, Kecskemét, Bács-Kiskun county | 22 | 12 |
| Borsod-Abaúj-Zemplén Megyei Kórház és Egyetemi Oktató Kórház, ICU, Miskolc, Borsod-Abaúj-Zemplén county | 25 | 20 |
| ESzSzK, Szent István Kórház, General Surgery Department, Budapest | 15 | 10 |
| Flór Ferenc Kórház, ICU, Kistarcsa, Pest county | 12 | 10 |
| Uzsoki Utcai Kórház, **General Surgery and Surgical Oncology Department, Budapest** | 17 | 15 |
| Semmelweis University, 1st. Department of Surgery, Budapest | 45 | 34 |
| Semmelweis University, Department of Anaesthesiology and Intensive Therapy, Budapest | 55 | 38 |
| Semmelweis University, Heart and Vascular Center, Department of Vascular Surgery, Budapest | 20 | 17 |
| **Szabolcs-Szatmár-Bereg Megyei Kórházak és Egyetemi Oktatókórház, ICU, Nyíregyháza, Szabolcs-Szatmár-Bereg county** | 30 | 22 |
| Szent János Kórház, ICU, Budapest | 25 | 11 |
| Vaszary Kolos Kórház, ICU, Esztergom, Komárom-Esztergom county | 5 | 5 |
| Vaszary Kolos Kórház, General Surgery Deparment, Esztergom, Komárom-Esztergom county | 10 | 9 |
| Zala Megyei Kórház, ICU, Zalaegerszeg, Zala county | 18 | 14 |

**Online:**

**Petz Aladár Megyei Oktató Kórház,** General Surgery Department**, Győr, Győr-Moson-Sopron county**

Semmelweis University, 2nd. Department of Surgery, Budapest

Semmelweis University, Department of Transplantation and Surgery, Budapest

University of Debrecen, [Department of Anestesiology and Intensive Care](http://aitt.med.unideb.hu/en), Debrecen, Hajdú-Bihar county

University of Debrecen, Department of Surgery, Debrecen, Hajdú-Bihar county

University of Pécs, Department of Anesthesiology and Intensive Therapy, Pécs, Baranya county

University of Pécs, Department of Surgery, Pécs, Baranya county

University of Szeged, Department of Anesthesiology and Intensive Therapy, Szeged, Csongrád county

University of Szeged, Department of Surgery, Szeged, Csongrád county
